# Blockchain-Enabled Asynchronous Federated Learning in Edge Computing

**DOI:** 10.3390/s21103335

**Published:** 2021-05-11

**Authors:** Yinghui Liu, Youyang Qu, Chenhao Xu, Zhicheng Hao, Bruce Gu

**Affiliations:** 1 School of Information Technology, Deakin University, Burwood, VIC 3125, Australia; liuyingh@deakin.edu.au; 2 Deakin Blockchain Innovation Lab, School of Information Technology, Deakin University, Burwood, VIC 3125, Australia; y.qu@deakin.edu.au (Y.Q.); xueri@deakin.edu.au (C.X.); 3 State Key Laboratory of Smart Tourism, Beijing Union University, Beijing 100101, China; 4 College of Engineering and Science, Victoria University, Footscray, VIC 3011, Australia; bruce.gu@vu.edu.au

**Keywords:** federated learning, blockchain, edge computing, asynchronous convergence

## Abstract

The fast proliferation of edge computing devices brings an increasing growth of data, which directly promotes machine learning (ML) technology development. However, privacy issues during data collection for ML tasks raise extensive concerns. To solve this issue, synchronous federated learning (FL) is proposed, which enables the central servers and end devices to maintain the same ML models by only exchanging model parameters. However, the diversity of computing power and data sizes leads to a significant difference in local training data consumption, and thereby causes the inefficiency of FL. Besides, the centralized processing of FL is vulnerable to single-point failure and poisoning attacks. Motivated by this, we propose an innovative method, federated learning with asynchronous convergence (FedAC) considering a staleness coefficient, while using a blockchain network instead of the classic central server to aggregate the global model. It avoids real-world issues such as interruption by abnormal local device training failure, dedicated attacks, etc. By comparing with the baseline models, we implement the proposed method on a real-world dataset, MNIST, and achieve accuracy rates of 98.96% and 95.84% in both horizontal and vertical FL modes, respectively. Extensive evaluation results show that FedAC outperforms most existing models.

## 1. Introduction

The fundamental technology of artificial intelligence (AI), machine learning (ML), has been the core drive force for the 4th industrial revolution [1]. An increasing number of data-driven application have been developed in many areas, e.g., the facial recognition model trained by convolutional neural network (CNN) has been widely applied for smartphone authentication [2], hospital utilizes regression model based on historical medical records to predict patient condition [3], and forthcoming autonomous vehicles. At the same time, the Internet of things (IoT) industry is also growing explosively. According to Lueth [4], by the end of 2020, there will be 21.7 billion active devices connected to networks all over the world, in which nearly 54% (11.7 billion) are IoT devices. By 2025, the number of IoT devices may raise to 30 billion. Consequently, these ubiquitous IoT devices generate a great amount of data day by day. With these growing trends, there will be an increasing number of applications driven by the generated data.

In the meantime, speedy growth of data science not only provides a great convenience to everyday life, it also brings corresponding issues and challenges, like the privacy leakage risk and data islands [5,6]. The largest data leakage scandal that happened in recent years is the Facebook–Cambridge Analytica data event, in which the UK consulting company, Cambridge Analytica, used millions of Facebook users’ data without user consent for political advertising purposes [7]. Subsequently, almost all fields of information technology highly related to data have received widespread attention, including data-driven machine learning technologies [8]. Thus, worldwide governments issued laws and regulations to protect individual privacy, i.e., the General Data Protection Regulation released by the EU and Cyber Security Law published by China [9]. As a result, personal data is protected effectively, while both the scale and difficulty of ML implementation with the conventional method are extremely restricted, because it is nearly an impossible mission to gain permission from millions of users. Moreover, individuals naturally expect that ML can train models securely, especially against specifically designed attacking techniques, such as inference attacks and poisoning attacks. Under this situation, in 2017, a privacy-preserving ML framework named federated learning (FL) was proposed by Google [10].

Classic FL is designed to work in a stable environment, such as a data center, in which network has high input-/output-speed and low latency [11,12]. Therefore, the convergence mechanism in classic FL is adopted in asynchronousmode [13]. The weakness is in each iteration, global model aggregation will be executed when it receives all local model updates, and once any worker somehow trains unsuccessfully, the training task may fail to converge [14]. Moreover, a central server in charge of global model convergence will be a potential risk node because of single-point failure [15], which means once this device has any problems, no other device can take over the duty of the center server by aggregating the global model [16]. Motivated by this, we propose an asynchronous FL convergence (FedAC) with staleness coefficient. In FedAC, the system will achieve local training and global convergence totally asynchronously, and errors that occur on any worker will not intermit the model training task. To further improve, we design a decentralized FL (FedBlock), in which the blockchain network is responsible to global model aggregation instead of a center server. It can enhance system robustness to avoid single-point failure and attacks aimed at the central node by adversaries. Through massive simulated experiments, the accuracy rate of prediction using the MNIST dataset are 97.45% in horizontal FL and 95.84% in vertical FL mode. The evaluation results show the superiority of the proposed model.

The main contributions of this work are summarized as follows.

- **Decentralized federated learning**: blockchain-enabled FL provides a decentralized global model convergence environment, in which all model updates will be verified by the blockchain consensus algorithm and stored on public ledgers in a decentralized way.
- **Efficient asynchronous convergence**: FedAC enables asynchronous local model training, updating, and global model aggregation. It can improve efficiency by avoiding the standby time of high-performance local devices.
- **Robust system**: By avoiding Single-Point Failures, model training process cannot be interrupted or suspended. Besides, blockchain provides extra secure protection for cyberattacks such as poisoning attacks.

The remainder of this paper is organized as follows. In Section 2, we first review existing research regrading FL optimization in recent years. In Section 3, we present the modeling and algorithm explanation of the proposed model.In Section 4, we continue to discuss experiment preliminaries, including setup of the physical environment, model parameters, and the blockchain. Besides, we state experiment results and a discussion. Finally, we conclude this paper in Section 5.

## 2. Related Works

In this section, we present relevant research on synchronous and asynchronous FL, edge FL, decentralized FL (including blockchain-enabled FL), heterogeneity, and communication Cost.

### 2.1. Synchronous and Asynchronous FL

In most ML fields, both synchronous and asynchronous were a core topic in terms of the learning process. For baseline FL, owing to Google scholars’ emphasis to cope with Non-IID and unbalance scenarios, in the original FL framework, they used FedAvg for convergence of model, which is a synchronous method [10]. However, in a real implementation environment, edge devices are not working in the data center, where massive unreliable factors may result in training faults, i.e., limited network bandwidth, power restriction. Moreover, the expectation of all edge devices successfully training in each round is impossible. Therefore, the asynchronous mechanism is usually considered to cope with parallel issues.

There are advantages and disadvantages in both algorithms. Synchronous aggregation is easy for deployment, and the global model converged from the edge local model is a serial-equivalent computation [17]. However, in synchronous mode, it is hard to deal with some harsh conditions. In comparison, it may be hard to deal with model aggregation using the asynchronous method if each edge node learning status is not synchronous [18].

Regarding asynchronous aggregation, several existing researches have been conducted. In [18], the authors proposed an asynchronous training method with dual-weights correction, the study status is a diverse situation in edge nodes. Another research [19] focused on vertical FL, and devised an algorithm to allow edge devices to execute the stochastic gradient method without communication with other devices. In [20], Mhaisen et al. proposed a semiasynchronous FL model that hierarchically trains a FL task in two phases including user-to-edge and edge-to-cloud. An adaptive FL model is proposed in [21], which considers the optimal trade-off between local training and global parameter aggregation in an asynchronous way to minimize the loss function under specific resource constraints.

### 2.2. Edge FL

There are some studies focused on federated edge computing. In [22], the authors proposed EdgeFed to apply local model updating to the edge server, which decreases computation cost and communication expense in the center data node. In [23], the authors designed a privacy-aware service placement (PSP) scheme in an edge-cloud system, which efficiently addressed individual privacy issues and provided better QoS to users. Additionally, approaches to improve edge FL can be achieved from clients side. In [24], the researchers proposed a novel framework, namely, FedCS, which deals with clients selection problems, resulting in a system aggregated with several more local updates and accelerating model performance advances. In [25], the authors apply local Differentially Private into clients, playing the role of protecting local privacy. For further improvement, the authors also proposed a randomly distributed update scheme to decrease security threats aimed at the center convergence node. Moreover, in [26], the authors implemented edge FL on vehicular edge network scenario and gained excellent performance. In [27], by deploying multiple deep reinforcement learning (DRL) agents, the authors optimized communication cost between IoT devices and edge devices. However, these aforementioned edge FL studies still adopt synchronous convergence strategy while the center server node plays the irreplaceable role of global model aggregation.

### 2.3. Decentralized FL

To address current issues of traditional FL, such as single-point failure, lack of incentive mechanism, and data falsification, blockchain-enabled federated learning [14,15,28,29,30,31] has been proposed and attracted massive attention from both academia and industry. In [15], the authors discussed the privacy issues of Blockchain-Enabled FL; further, a game-based model using the Markov decision process for industrial 4.0 was proposed in [28]. In [29], the authors devised a novel consensus algorithm specially designed for Blockchain-Enabled FL to improve the performances. Some researchers also discussed the application of Blockchain-Enabled FL into vehicular networks [30,31]. More and more relevant researches are emerging in various scenarios.

Despite a remarkable characteristic of FL being decentralized training, there is still a demand on a trusted third party for the role of coordination, which could be a single failure node in the network. Therefore, for the purpose of improving FL system robustness, a strategy integrating blockchain technology is proposed, which achieved decentralization completely [32]. By adopting the nature of blockchain, a consensus mechanism, [33] accomplished global model convergence without center server. The weakness is that this end-to-end switch mechanism may lead to extra communication cost in the network. The search [33] contributed by reducing samples on edge device to accelerate training locally. Future studies should focus on coping with latency and communication load reduction in blockchain-based FL.

### 2.4. Heterogeneity and Communication Cost

The heterogeneity related to Non-IID data, unbalanced data, various hardware performance of devices, edge devices operation system, wireless network environment, etc., are challenging FL evolution. Some related works coped with these issues by fair resource allocation, convergence updating, fault tolerance, and personalization of the edge device training mode. The study [34] designed an incentive algorithm leading to high-quality data holders that are more willing to participate in model training, accordingly, to promote model accuracy. The research [35] stated a novel method working on Non-IID data, which, through computing the aggregation bound, decreased loss function under restricted resource. Bonawitz [36] coped with training issues of abnormal suspends via discarding a network-disconnected mobile device, caused by a poor wireless network or power constraints. The study [37] clarified a novel algorithm, federated augmentation (FAug), via augmenting missing data in each device to transform Non-IID data into IID data, resulting in improving model accuracy by 95–98%.

Communication Cost is the major factor that may influence the model performance, in other words, it could be the bottleneck [36]. Even if the traffic in network was only gradient updates rather than the entire model, consideration of the network scale may involve millions of edge devices. Limited bandwidth in the mobile network and slower communication speed may also fall below expectations. Two technology points can be considered for dampening network pressures: (1) decreasing gradient updates size, i.e., using a more efficient compression algorithm; (2) optimizing correspondence rounds to an appropriate value. Currently, there are couples of researches contributing to communication optimization. Caldas [38] introduced a lossy algorithm for model transmitting, which drops network loads originated from the server 14 times and communication cost from clients 28 times. The research [10], through increasing batch size, avoided frequent communication rounds and achieved a great result.

From the above literature analysis, it can be concluded that FL has been and is still experiencing a fast boom and improvement in various aspects. However, a decentralized and asynchronous FL that is efficient and robust with high accuracy has barely been discussed.

## 3. System Modeling

In this section, we demonstrate the formulation of federated learning asynchronous convergence (FedAC) considering the staleness coefficient and Blockchain-Enabled federated learning (FedBlock).

### 3.1. FedAC with Staleness Coefficient

In this context, the linear regression task to be solved by edge devices is shown as Equation (1).

(1) $$\min_{\omega }f\left(\omega \right)=\sum_{i=1}^{K}\sum_{j=1}^{R}\zeta_{i}^{j}f\left(\omega_{i}^{j}\right),$$

where *K* denote total numbers of edge devices: K:={1,…,k∈K} with |K|=k. A dataset of *D* samples, where $D=\cup_{i=1}^{K}d_{i}$, is maintained by *K* edge devices. *R* is defined as the total number of training iteration rounds, R:={1,2,…,r∈R} with |R|=r. The *i*-th miner $m_{i}$ associated device $k_{i}$, instead of a fixed center server, is selected randomly from a series of miners M:={1,…,m∈M} with |M|=m. Moving on, to solve the training and update delay caused by asynchronous training, we define the staleness coefficient ζ to decrease latency device contribution in a global model. In a new iteration *r*, as the delayed device $k_{i}$ has gained the up-to-date global model from its associated miner $m_{i}$, value $\zeta_{i}^{r}$ will be computed automatically by comparing the updated version from the global model. Therefore, for the iteration *r*, $\zeta^{r}$ is defined as Equation (2).

(2) $$\zeta^{r}=\sum_{i=1}^{K}\zeta_{i}^{r}=1\;\left(0<\zeta_{i}^{r}\leq 1\right).$$

Moving on, The *i*-th edge device’s local function $f_{i}$ is parameterized by $\lambda (\omega ;x_{i},y_{i})$, where λ is the predefined loss function operated with data point $\left\{x_{i},y_{i}\right\}$. In the data point, $x_{i}$ belongs to a *d*-dimensional column vector with $x_{i}\in R^{d}$, while $y_{i}$ is respected to be a scalar value, where $y_{i}\in R$. In this paper, we use LogSoftmax and NLLLoss as loss functions to cope with the multiclassification task. $\nabla f_{i}\left(\omega_{i}^{t}\right)$ means the gradient of $k_{i}$ device in *r*-th training round and δ is the learning rate. The device $k_{i}$ locally trains the model using local data sample $d_{i}$, adopting the stochastic variance reduced gradient (SVRG) method. The model parameters are computed as Equation (3).

(3) $$\omega_{i}^{r+1}=\omega_{i}^{r}+\delta \nabla f\left(\omega_{i}^{r}\right).$$

Furthermore, edge device $k_{i}$ uploads the trained local model to the miner $m_{i}$, who aggregates the global model with *r*-th round staleness coefficient $\zeta_{i}^{r}$, updated local weight $\omega_{i}^{r}$, and global model weight $\omega_{global}$ newly updated by device $k_{\left(i-1\right)}$. The formulation is shown as Equation (4).

(4) $$\omega_{global}^{r}=\left(1-\zeta_{i}^{r}\right)\omega_{global}+\zeta_{i}^{r}\omega_{i}^{r}.$$

After that, the up-to-date aggregated global model will be downloaded by all associated edge devices for training of the next iteration. Unlike conventional FL, for FedAC, a center server that is responsible for aggregating the global model is replaced by the miner randomly selected by the consensus process of blockchain. However, all local training and global aggregation tasks will be executed repeatedly until the global model satisfies predefined constraints as

(5) $$|\omega_{global}^{r}-\omega_{global}^{r-1}|\leq \tau ,$$

where τ>0 is a small positive constant. Additionally, the tasks will be forced to quit if all training iterations are completed. The FL procedure is shown in Algorithm 1.
**Algorithm 1** FedAC
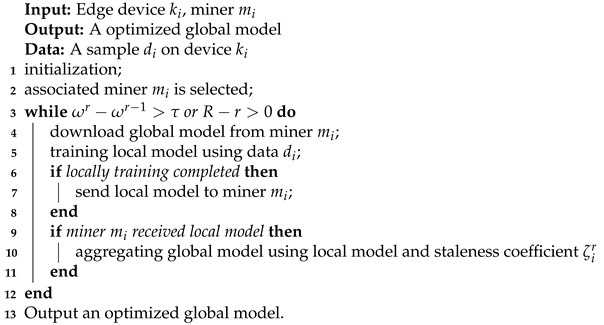


### 3.2. Decentralized Federated Learning Using Blockchain (FedBlock)

In order to exchange model parameters truthfully, FedBlock generates blocks and conducts cross-verification on model parameters while saving them on a distributed ledger. Each block in the public ledger includes both a header and a body sector. For conventional blockchain, the body normally stores a specific number of transactions verified by miners, while in FedBlock, it contains the updated model parameters from device $k_{i}$, i.e., {$\omega_{i}^{r},\nabla f\left(\omega_{i}^{r}\right)$} for device *k* in round *r*, and also the computation time $T_{i}^{r}$ of device $k_{i}$. The header part is designed to include the information of a pointer to the previous block, block generation rate β, and the output value (nonce in this context) of the consensus method (Proof-of-Work (PoW)). For the purpose of storing all local updated model parameters, the formulation of block size is designed as Equation (6), where *S* is defined as block size, *h* as the header size, and $\alpha_{m}$ as the updated local model size.

(6) $$S\leftarrow h+\alpha_{m}K.$$

The miner $m_{i}$ is designed to produce a candidate block, which involves updated local model information from associated edge devices or other miners. The stored procedure will run persistently until the block size is fully occupied or the waiting time $T_{wait}$ expires. In order to ensure each block is written with the local updated model, $T_{wait}$ must be sufficiently long.

For the consensus process, the miner $m_{i}$ will continue to generate a random hash value until it becomes smaller than a target value (nonce). Once $m_{i}$ works out the nonce value, the candidate block in $m_{i}$ is regarded to be a new block. Similarly, the block generation rate β can be controlled carefully by changing the difficulty coefficient of the PoW consensus algorithm.

The up-to-date-released block is sent to all miners in a broadcast manner for the synchronization of all distributed ledgers. For this aim, all miners receiving the up-to-date block will be enforced to exit the consensus computing session and append the block to corresponding local ledgers. Nonetheless, a situation may occur where another miner $m_{i+1}$ also generates a candidate block within a negligible time slot, but other miners deny to append that block due to receive delay. In FedBlock, forking may result in edge devices receiving a set of false global model parameters for the next training iteration, and subsequently generate incorrect local model updates in the following rounds.

The blockchain generation rate β and the block linking delay will be associated with forking frequency positively. We discuss the time consumption regarding the mitigation of forking in the following parts.

In addition to the previously discussed actions to update local trained models, FedBlock offers both data rewards to edge devices and mining rewards to global aggregation as well as producing the candidate block. The edge device $k_{i}$ receives data rewards from its associated miner $m_{i}$, and the amount of the rewards is set to be proportional to the size of data sample $d_{i}$. The miner $m_{i}$ will also gain the mining rewards from FedBlock, such as data rewards; mining rewards are also linearly proportional to the convergence size of data samples associated with edge devices, that is, $\sum_{i=1}^{k_{m}}$, where $k_{m}$ means all associated edge devices with miner $m_{i}$. However, FedBlock is able to offer an incentive to miners since miners can operate as many local training models as possible and offset the cost on data rewards at the same time.

**Figure 1:**
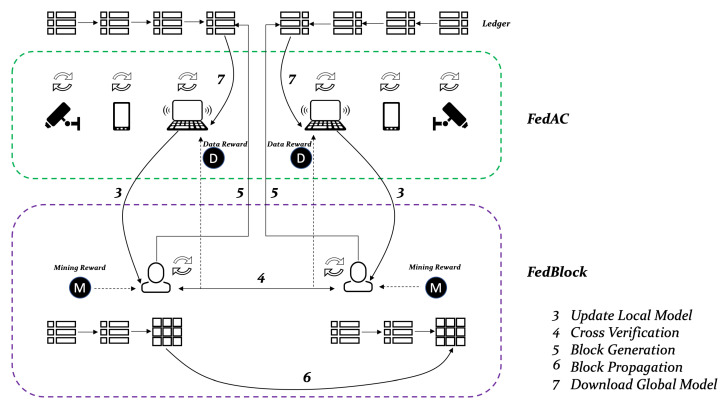
Workflow of FedAC and FedBlock.

The Figure 1 illustrates the structure of both FedAc and FedBlock. To better clarify, we use Algorithm 2, which consists of eight processes, to explain the workflow of FedBlock.

In the initialization stage, when the aforementioned miner selection process is completed, the blockchain network will generate a global model, which uses a range of weight values that satisfy Equation (7). Then, device $k_{i}$ bonded to the miner $m_{i}$ downloads the initial global model $f(\omega_{global}^{0})$ for local training.

(7) $$\begin{array}{c} \omega_{global}^{0}\in \left(0,\omega_{max}\right)\;\mathrm{and}\;\nabla f\left(\omega_{global}^{0}\right)\in \left(0,1\right]. \end{array}$$

In the local training stage, all edge devices *K* will update their local models, adopted in Equation (3), using the global model downloaded from the blockchain network and the locally-held data sample.
**Algorithm 2** FedBlock
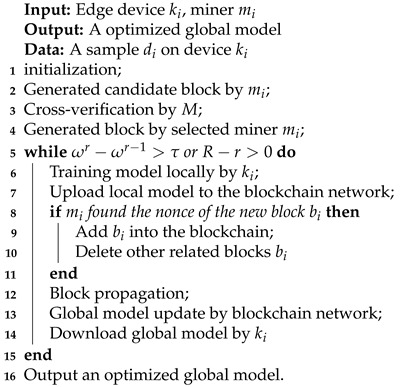


In the model upload stage, once an edge device $k_{i}$ finishes its training locally, it uploads both the local model parameters {$\omega_{i}^{r},\nabla f\left(\omega_{i}^{t}\right)$} and local computation time $t_{i}^{r}$ to its associated miner $m_{i}$.

In the cross-verification stage, each miner $m_{i}$ will share the uploaded local model via the blockchain network. Meanwhile, miners will verify the received local model updates or the other miners in order of arrival. If the local computation time $t_{i}^{r}$ is proportional to a device’s training data sample $d_{i}$, the truthfulness of local model updates can be validated. Then, the verified local model updates will be recorded in the candidate block of miner $m_{i}$ until the size limit $S=h\alpha_{m}K$ is reached or waiting time $T_{w}ait$ expires.

In the block generation stage, all miners will work with a consensus algorithm to find the nonce value or receive a candidate block from another miner.

In the block propagation stage, the miner who is first to find the nonce is denoted as $m_{\phi }\in M$. This miner will generate a candidate block as a new block that will be shared with other miners. In FedBlock, the acknowledgment (ACK) mechanism is applied for avoiding forking events. All miners will wait to receive an ACK signal from other miners; otherwise, the process loops back to stage 2.

In the global model update stage, the edge device $k_{i}$ will execute global model aggregation as Equation (4) using the local model updates stored in the generated block.

In the global model download stage, the edge device $k_{i}$ will download the candidate block with global model parameters from the blockchain network.

The total processes will work repeatedly until the global model satisfies $|\omega_{global}^{r}-\omega_{global}^{r-1}|\leq \tau$ or r>R.

The complete notation description refers to Table 1.

## 4. Evaluation and Experimental Preliminaries

In this section, we first simulate an edge computing environment, including physical devices, real-world datasets, and FL model configuration. Then, based on this environment, we implement experiments to show the performances of the proposed model. We provide experimental results derived from various configurations, including different numbers of edge devices, data distribution, learning rate, and discuss accuracy, convergence, and time consumption, respectively.

### 4.1. Physical Environment Deployment

**Figure 2:**
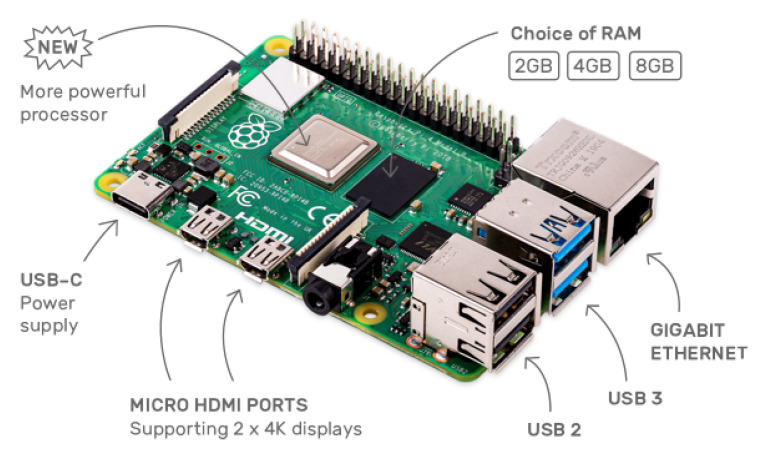
Raspberry Pi (4b) diagram.

There are several capable single-board computers that can be selected currently, i.e., Raspberry Pi (4b), Nvidia’s Jetson Nano Developer Kit, Banana Pi M3, or NanoPi NEO3. For a machine learning task, Nvidia’s Jetson Nano Developer Kit might be the best one in the above choices because it is naturally designed for edge computing. However, with considerations of performance, extensions, cost, and availability of affluent development references, we selected the Raspberry Pi (4b), which is shown in Figure 2. For the simulation of edge devices and miners, Raspberry Pi (4b) is a tiny single-board computer in a credit card size but has powerful computation ability and a diverse range of extension ports with abundant input and output options, e.g., Wi-Fi, LAN, Bluetooth, USB, Audio, Video, and HDMI. Especially, it provides general-purpose input–output (GPIO) connectors that can expand extra sensors for more input- and output-demands. In order to reflect the diversity of edge devices, we selected 3 kinds of memory sizes: 2 GB, 4 GB, and 8 GB, respectively. Specifications of the Raspberry Pi (4b) are shown in Table 2.

Furthermore, with consideration of avoiding network connectivity issues resulting in the failure of experiments, we assume the network environment is stable. Thus, we use a network switcher with Gigabit Ethernet and the port type is 1000BASE-T, which follows the standard of IEEE 802.3ab (twisted-pair cabling i.e., Cat-5, Cat-5e, Cat-6, Cat-7, supporting up to 100 m).

### 4.2. Federated Learning

To test and verify the performance of the model in real-world scenarios, in this context, we apply the classic dataset MNIST for all tasks, and dataset CIFAR-10 for 6 edge clients. MNIST is a subset of NIST, which is the dataset of handwriting images for the purpose of image recognition machine learning training, including 60,000 28 × 28 pixels examples for training and 10,000 28 × 28 pixels examples for testing. The CIFAR-10 dataset consists of 60,000 32 × 32 color images in 10 classes, with 6000 images per class. There are 50,000 training images and 10,000 test images.

To best simulate a wide range of application scenarios, we considered several federated learning features as follows.

- Non-IID: The data held by some particular devices have specific features that do not exist on the majority of devices.
- In order to protect user privacy, federated learning is adopted as the distributed computation framework for federating data holders. This may lead to a larger number of participants than classic machine learning.
- Data size imbalance: Due to the heterogeneity of training devices and differences of working environment, some devices may possess more examples, while others hold less.
- Limited resources and poor network quality: The first constraint is also due to the heterogeneity of devices. Besides, in real edge environment, edge devices may work in unstable and unreliable networks, i.e., mobile phones may go offline frequently due to a variety of reasons.

In a real-world environment, edge devices include massive smart embedded devices (e.g., smartphones, cameras, sensors, or even autonomous vehicles). The data generated by edge devices are usually Non-Independent and -Identically Distributed (Non-IID) and distributed in an unbalanced manner on each data holder. That means the data processed by each edge device has unique features. This is also considered and named as vertical FL. Therefore, we adopt two kinds of data distribution in experiments. The first one is we assume that training conditions are ideal, i.e., the devices working in edge environments are simple, the types of devices are similar, and data distribution is also homogeneous, which means most devices possess data with similar features. For this scenario, all participating devices hold similar numbers of examples and almost all features. This can be considered as horizontal federated learning. The second one is that the devices are working for diverse aims in edge environments, which will lead to unbalanced distribution of features on each device. The most extreme situation is that each device possesses unique features that cannot be found in other devices. In this context, the distribution of data is designed so that each participant just holds unique features. This is also known as vertical federated learning.

The model we implemented in the experiments is the classic CNN. With consideration of edge devices’ computation ability and characteristics of the datasets, we use a typical CNN configuration, which contains two convolution layers, including 20 × 5 and 50 × 5 channels. Data outputted from each convolution layer will be activated by the ReLU function, then pooled by the Max_Pool function. In addition, the channels are connected by two fully-connected layers, including 800 and 500 units. Between both fully-connected layers, data is activated again by ReLU. Finally, 10 units are outputted by the Log_Softmax function.

To mitigate the complexity of global convergence in asynchronous situations, we use the single epoch model instead of multiple epochs. For the training round, the minibatch was 32, whereas 128 was set up for testing. The learning rate has two options, which are fixed mode and decay mode. In fixed mode, the default value is 0.1. We may fine-tune the learning rate for model optimization. For decay mode, the learning rate is decreased by 2% in each new round of iteration.

However, for the reason that the procedures of training, updating, and aggregation on each device are totally asynchronous, we employ a method to test the model periodically, while time consumption will be computed until the last device finishes its task.

### 4.3. Accuracy Evaluation

**Figure 3:**
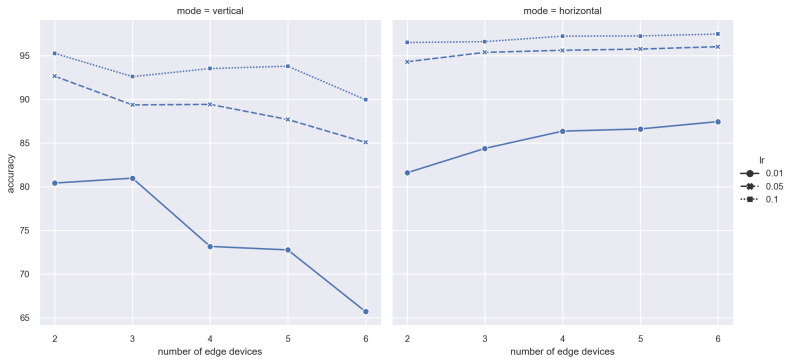
Accuracy vs. number of Edge devices.

To start with, we set the training batch as 32; training round as 200; and learning rate as 0.1, 0.05, and 0.01, where the learning rate will decay 2% in each new training iteration. We implement experiments on vertical and horizontal FL models, respectively. As shown in Figure 3, when the learning rate equals 0.1, all tests (2, 3, 4, 5, 6 devices) achieve accuracy rates of over 90% in both vertical and horizontal FL modes. For vertical FL, the highest value, 95.25%, is obtained when the settings are 2 edge devices and lr = 0.1. Regarding horizontal FL, the highest accuracy rate is 98.68% when there are 6 workers and lr = 0.1. On the other hand, for MNIST dataset, the appropriate learning rate is 0.01. By comparison, when the learning rate is set as 0.01, both vertical and horizontal FL never reach an accuracy rate of 90%. Moreover, because each worker possesses unique features that are not held by other devices, in vertical FL, more edge devices participating will result in lower accuracy. Up to a 55% decrease of accuracy is verified in highly skewed Non-IID data distribution [39]. By contrast, in horizontal FL, an increasing number of workers participating in local training will help gain a better global model. However, the situation that each participating worker just holds unique features is an extreme case. In real-world scenarios, the most common scenario is that each kind of featured data is maintained by multiple devices.

**Figure 4:**
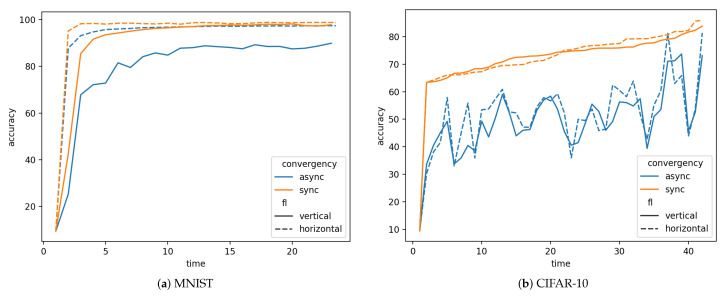
Asynchronous vs. Synchronous.

As shown in Figure 4, for the MNIST dataset, both asynchronous and synchronous modes reach near 98% accuracy in horizontal FL while asynchronous mode achieves 89.94% and synchronous mode gains 97.86% in vertical FL. For dataset CIFAR-10, synchronous mode gets a little bit higher accuracy than asynchronous mode, which are 85.92%, 81.37% in horizontal FL and 83.83%, 73.12% in vertical FL, respectively.

### 4.4. Convergence Evaluation

**Figure 5:**
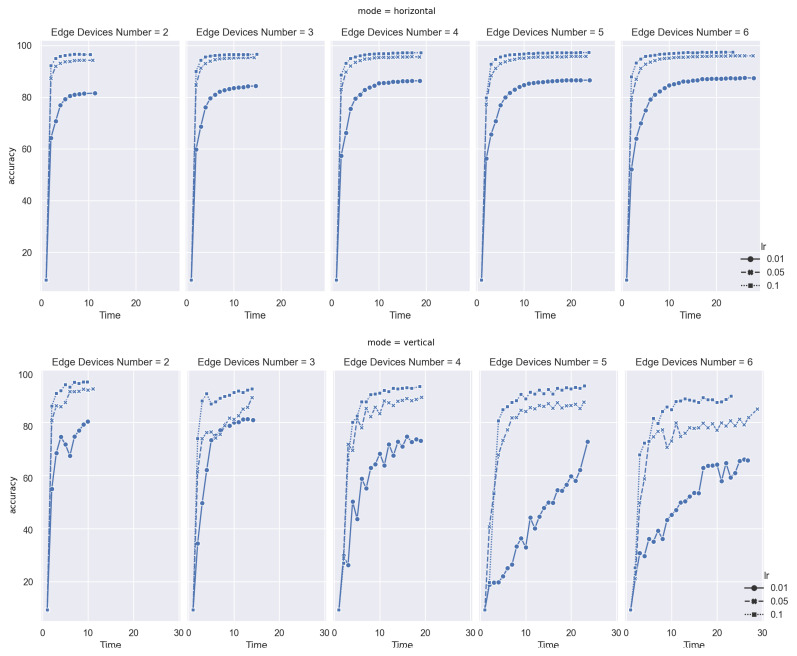
Convergence on Horizontal and Vertical FL in MNIST.

As shown in Figure 5, in given training rounds, all tasks achieve a convergence, and even performance on each scenario is diverse. In the following part, we will discuss these cases in detail.

Firstly, when the learning rates are 0.1 and 0.05, the convergence is rapidly reached at an accuracy of more than 90% in nearly 10 min when 2 devices are involved. The most significant difference is in horizontal FL, where the curve of aggregation is smooth, whereas it fluctuates in vertical FL because discrete feature distribution leads to a deviation in global model weight. This situation is even significant on asynchronous modes. For example, in the case when learning rate is 0.01 and device number is 4, the curve moves like a wave and keeps climbing up.

Furthermore, a unique feature exists in vertical FL mode; that is, nearly all curves having a distinct improvement near the training finish stage. The most obvious one is when the device number is 5 and the learning rate equals 0.01. Due to the heterogeneity of each edge device, local training time may significantly vary. For synchronous mode, in each round of global model aggregation, the miner waits for the last worker’s training updates before aggregation, so the delay among each device is not obvious.

However, for asynchronous mode, this delay will increasingly accumulate, so weights in the global model will be more and more deviated to devices with high performances. When the task reaches the final stage, the global model will be balanced by converging lagging local model update gradients. By contrast, in the horizontal FL model, the curve is much smoother when the task is nearly completed.

Despite that FedAC is not designed to cope with Non-IID issues specifically, it can work with both FL modes and have greater outputs regarding convergence.

### 4.5. Time Consumption Evaluation

In a training round, the time cost $T_{total}$ includes local training time $T_{local}$, global convergence time $T_{global}$, and time cost of blockchain $T_{blockchain}$. Therefore, in this project, overall task time consumption is a unit time cost multiple of the training rounds. The formulation can be indicated as Equation (8).

(8) $$T_{total}\leftarrow rT_{local}+rT_{global}+rT_{blockchain}.$$

**Figure 6:**
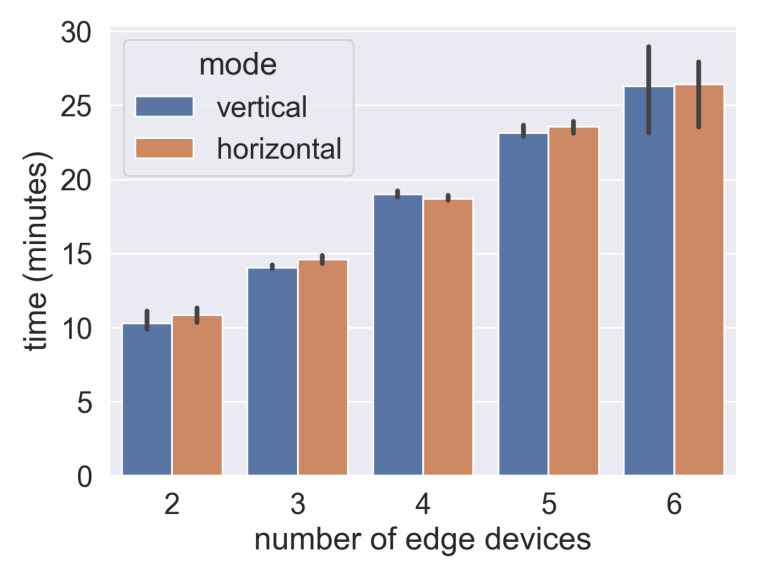
Time Consumption.

As shown in Figure 6, wherever in vertical and horizontal FL, to give a constant training round, the time cost increases with the increasing number of edge devices participating. Indeed, in asynchronous mode, global model convergence does not concern single device performance, but for Non-IID data, less updating means that the global model may include fewer weights working on this device-held feature data. Thus, in this experiment, we set up testing wait for all workers’ tasks done. That is why total time costs are similar for both vertical and horizontal FL. As Figure 5 and Figure 6 illustrate, total time cost in the scenario with 6 devices is nearly 2 times the scenario with 2 workers. This is a significant point varied with conventional machine learning, in which a growing trend of participants may accelerate model training. However, communication cost is a major bottleneck for future FL development. An idea in terms of reducing time consumption is to reduce communication rounds, i.e., increasing batch size for a training iteration or set a converging global model after multilocal training instead of every round aggregation.

### 4.6. Consensus of Blockchain Evaluation

**Figure 7:**
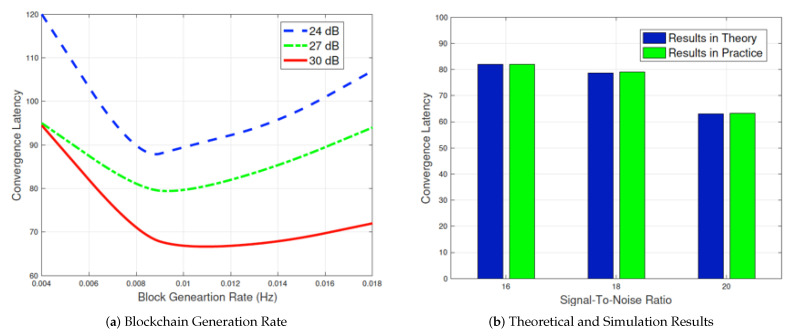
Blockchain evaluation regarding blockchain generation rate.

In this subsection, we show how blockchain generation rate β influences the convergence latency of the proposed model in Figure 7. We can observe that the convergence latency of the proposed model is shown as a convex curve over the blockchain generation rate β. From the second figure, it can be observed that the convergence latency decreases with the increase in the signal-to-noise ratio (SNR). If we define β∗ as the optimal generation rate, the minimum convergence latency can be obtained based on it. Although the latency value of the simulated results is 1.8% higher than the results derived from theory, the performance is still comparable and testifies to the feasibility of the proposed model.

## 5. Summary and Future Work

In this paper, we propose advanced FedBlock and FedAC models for a decentralized and asynchronous federated learning framework. FedBlock enables decentralized FL built upon blockchain while FedAC allows the FL to conduct global aggregation in an asynchronous manner considering a staleness coefficient. The proposed framework is robust to various security threats such as poisoning attacks and single-point failures while being efficient due to the asynchronous aggregation. The simulation results show that the performance of the proposed framework is comparable to existing synchronous FL while having an optimal block generation rate of the blockchain consensus process.

For future works, we will focus on larger distributed devices scenarios. More edge devices participating means more undiscovered factors, which may influence the training process and model accuracy. In addition, the topic of Non-IID optimization is also a large challenge due to the fact that data distribution in the real-world is heterogeneous and unbalanced. Furthermore, privacy issues of blockchain-enabled federated learning will be addressed using differential privacy or other advanced techniques.

## Figures and Tables

**Table 1:** List of Notations.

Notation	Description	Notation	Description
*K*	Total number of edge devices	λ	Federated learning loss function
$k_{i}$	The *i*-th edge device	ζ	Staleness coefficient
*M*	Total number of miners	δ	Federated learning learning rate
$m_{i}$	A miner associated with edge device $k_{i}$	*x*	*d*-dimensional column vector
*D*	Sample space	*y*	A scalar value
$d_{i}$	A subset of the sample space on edge device $k_{i}$	$\left\{x_{i},y_{i}\right\}$	*x* and *y* on edge device $k_{i}$
*R*	Total number of training rounds	τ	A small positive constant
*r*	The *r*-th round	*T*	Time
f(ω)	Final global model	$T_{wait}$	Waiting time
$f(\omega_{i}^{r})$	Local model on edge device $k_{i}$ in round *r*	β	Block generation rate
∇f(ω)	Gradient	*S*	Block size
ω	Model weights	*h*	Head size in block
$\omega_{i}$	Edge device local model weights	α	Updated local model size in block

**Table 2:** Specifications of Raspberry Pi (4b).

Items	Description
CPU	Broadcom BCM2711, Quad core Cortex-A72 (ARM v8) 64-bit SoC @ 1.5 GHz
Memory	2 GB, 4 GB or 8 GB LPDDR4-3200 SDRAM (depending on model)
Wireless LAN	2.4 GHz and 5.0 GHz IEEE 802.11ac wireless, Bluetooth 5.0, BLE
LAN	Gigabit Ethernet
USB	2 USB 3.0 ports; 2 USB 2.0 ports.
GPIO	Standard 40 pin GPIO header
HDMI	2 × micro-HDMI ports (up to 4kp60 supported)
Display	2-lane MIPI DSI display port
Camera	2-lane MIPI CSI camera port
Audio	4-pole stereo audio and composite video port
Video	H.265 (4kp60 decode), H264 (1080p60 decode, 1080p30 encode)
Graphics API	OpenGL ES 3.0 graphics
External Storage	Micro-SD card slot for loading operating system and data storage
USB-C Power	5V DC via USB-C connector (minimum 3A)
GPIO Power	5V DC via GPIO header (minimum 3A)
Power over Ethernet (PoE)	enabled (requires separate PoE HAT)
Working Temperature	Operating temperature: 0–50 degrees C ambient

## Data Availability

Two public datasets are used in this paper, which are MNIST (http://yann.lecun.com/exdb/mnist/, accessed on 20 February 2021) and Cifar-10 (https://www.cs.toronto.edu/~kriz/cifar.html, accessed on 20 February 2021).
